# Exploration of Targeted Anti-tumor Therapy: a contribution to the development of targeted therapies

**DOI:** 10.37349/etat.2020.00001

**Published:** 2020-02-29

**Authors:** Nicola Normanno, Graham Packham

**Affiliations:** 1 Cell Biology and Biotherapy Unit, Istituto Nazionale Tumori-IRCCS-Fondazione G. Pascale, 80131 Naples, Italy; 2 Cancer Research UK Centre, Cancer Sciences, Faculty of Medicine, University of Southampton, SO16 6YD Southampton, UK; University of Southampton, UK

When we were invited to become Editors-in-Chief of Exploration of Targeted Anti-tumor Therapy (ETAT), we both had the same question: do we need another new journal?

The number of journals in the field of cancer research has grown enormously in recent years and every researcher receives requests to submit papers to journals that promise rapid review and publication times, low costs, wide publicity of the publications, etc. With this wide field it can be a challenge for researchers is to identify the most suitable journal for their papers. So why did we agree to share this task of launching a new scientific journal?

After years of substantial progress, it is now clear that the successful development of target therapies requires co-ordinated input at all stages of the process from identification of new drug candidates through to development of appropriate diagnostic techniques and their clinical validation. This will continue to be important for future advances where attention is increasingly turning to novel targets and pathways, particularly in the setting of immunotherapy. The discovery of the mechanisms underlying tumor growth, the availability of technologies for the study of ever more sophisticated genetic-molecular alterations of the tumor and the development of increasingly powerful and selective inhibitors, is making it possible to introduce into clinical trials and clinical practice numerous novel targeted drugs.

So what convinced us was the potential for ETAT to develop a new multidisciplinary offering which will appeal to all members of the scientific community involved in the development of targeted therapies from chemists through to clinical researchers. The journal’s breadth is nicely illustrated by the contents of this first issue. Two review articles are focused on new possible targets for therapeutic approaches, mRNA translation initiation factors and the Fanconi Anaemia pathway. A further review addresses the issue of genetic markers currently in clinical use or in development in clinical trials for patients with metastatic colorectal cancer. Three papers cover different aspects, from pre-clinical research to clinical trials, which need to be closely integrated to guarantee the progress of targeted therapies.

We hope you will enjoy reading these and future articles and look forward to receiving your contributions.

## Abbreviations

ETAT: Exploration of Targeted Anti-tumor Therapy

## Declarations

### Copyright

© The Author(s) 2020.

